# Non-invasive Prediction of Lung Cancer Histological Differentiation *via* Radiomics and Multi-binary Classification Models

**DOI:** 10.2174/0115734056415019251016111351

**Published:** 2025-10-27

**Authors:** Haoran Jiang, Beichuan Zhu, Lin Xia, Yingying Han

**Affiliations:** 1 School of Health Science and Engineering, University of Shanghai for Science and Technology, Shanghai, China; 2 Department of Medical Affairs, People’s Hospital of Yangzhong City, Yangzhong, Jiangsu, China; 3 Department of Pathology, People’s Hospital of Yangzhong City, Yangzhong, Jiangsu, China

**Keywords:** Radiomics, Machine learning, Lung cancer, Histological differentiation, SHAP, NSCLC

## Abstract

**Background:**

The histological differentiation of Non-Small Cell Lung Cancer (NSCLC) is a critical prognostic factor that influences therapeutic strategies and patient outcomes. However, conventional assessment methods relying on postoperative pathology or biopsy are invasive and limited by sampling bias. Therefore, it is of great clinical significance to develop a non-invasive, imaging-based approach for accurate preoperative differentiation evaluation.

**Methods:**

This retrospective study included 184 NSCLC patients with preoperative chest CT scans and confirmed pathological differentiation grades from 2022 to 2024. Radiomics features were extracted using PyRadiomics, followed by feature selection *via* the LASSO algorithm. A novel three-task binary classification strategy was proposed to replace conventional trinary classification, including low *vs*. non-low, moderate *vs*. non-moderate, and high *vs*. non-high differentiation. Four machine learning models-GBDT, RF, XGBoost, and LightGBM-were constructed and evaluated using ROC analysis, confusion matrices, and SHAP-based interpretability analysis.

**Results:**

The GBDT model achieved the highest AUC (0.849) in the low differentiation classification task, while the RF model outperformed others in predicting high differentiation (AUC = 0.7188). The moderate differentiation task showed relatively poor performance across all models (AUC < 0.55). SHAP analysis revealed that features such as original_firstorder_Kurtosis, glrlm_RunEntropy, and wavelet-HLL_firstorder_Median played key roles in differentiating tumor grades, highlighting their biological relevance and potential utility in clinical interpretation.

**Discussion:**

The proposed multi-binary strategy improved classification granularity and interpretability. Ensemble learning models demonstrated robust performance across tasks, especially for extreme differentiation levels.

**Conclusion:**

This study, which combines radiomics with a multi-task machine learning framework, demonstrates prediction and can improve the accuracy and interpretability of preoperative lung cancer differentiation. The proposed model provides a non-invasive, quantitative tool with the potential to support individualized clinical decision-making. Further multicenter validation and multimodal data integration are warranted to enhance its clinical applicability.

## 1 INTRODUCTION

Lung cancer is the leading cause of cancer-related death worldwide, with Non-Small Cell Lung Cancer (NSCLC) accounting for the vast majority of its incidence. As a highly heterogeneous malignant tumor, the clinical behavior, prognostic assessment, and treatment decisions of NSCLC are closely dependent on the histological differentiation of the tumor [1-4]. Previous studies have shown that the higher the tumor differentiation level, the closer its cell structure is to normal lung tissue, the milder the clinical manifestations, and the better the response to treatment; while poorly differentiated tumors usually indicate high invasiveness and poor prognosis [5]. Currently, differentiation grade assessment is mainly based on pathological analysis of tissue samples obtained after surgery or puncture, but this type of method faces many challenges in clinical practice, including the invasiveness of the examination method, the lack of sample representativeness, and the consistency bias caused by subjective interpretation, which restricts its widespread application in preoperative classification and individualized treatment [6-8].

With the development of imaging technology and artificial intelligence algorithms, radiomics-as an emerging quantitative imaging analysis tool-provides the ability to automatically extract a large number of potential features that represent tumor heterogeneity from medical images [9-11]. A large number of studies have confirmed that radiomics can assist in tasks such as tumor classification, prognosis prediction, and treatment response evaluation, providing effective support for precision medicine [12]. However, in terms of predicting the degree of differentiation of lung cancer, most current radiomics studies still use the traditional three-classification strategy, which divides tumors into three categories: poorly differentiated, moderately differentiated, and well differentiated. Due to the overlap of imaging features between these three categories, the model lacks the ability to discriminate at the classification boundary, which is prone to category confusion, affecting the accuracy of the final prediction and clinical usability [13].

In addition to the limitations of classification performance, existing imaging omics models generally have the “black box” problem, which makes it difficult for doctors to understand the decision-making basis of the model, hindering its promotion and application in real clinical environments [14, 15]. Especially when it comes to key decision-making scenarios such as tumor differentiation, the transparency and interpretability of model predictions are particularly important. In recent years, the SHAP (Shapley Additive Explanations) method, as an interpretable algorithm based on game theory, has gradually been introduced into the imaging omics modeling process [16, 17]. It can not only provide a feature contribution score to individual prediction results, but also reflect the importance of global features, which helps to identify imaging features that are closely related to the degree of differentiation. This explanatory ability is of great significance for exploring the potential association between imaging features and biological behavior.

In response to the above challenges, some scholars have tried to circumvent the problem of blurred category boundaries by task reconstruction in recent years, among which the “multiple binary classification tasks” strategy is considered to be an effective alternative. This strategy divides the original multi-classification task into several independent binary classification models, which helps to enhance the ability of each model to distinguish specific categories, thereby improving the overall discrimination performance and model stability. In addition, this strategy also makes it easier to nest subsequent explanatory analyses in more specific classification scenarios, revealing the relationship between features and pathological phenotypes from different perspectives. In this context, this study proposed a new strategy for lung cancer differentiation prediction based on radiomics and machine learning algorithms. We retrospectively included preoperative CT imaging data, extracted high-dimensional radiomics features through PyRadiomics, used the LASSO method for feature screening, and constructed three independent binary classification models for identifying poorly differentiated, moderately differentiated, and well-differentiated tumors. We further used machine learning models such as GBDT, XGB, RF, and LGBM for performance comparison, and introduced the SHAP analysis framework to visualize the contribution of key features of each model.

## 2 METHODS

### 2.1 Patients

This study was a single-center, retrospective cohort study, and the data were derived from the imaging database of Yangzhong People's Hospital, Jiangsu Province. The study was approved by the Medical Ethics Committee of Yangzhong People's Hospital (ethics approval number: LW2023026), in accordance with the relevant provisions of the Declaration of Helsinki, and all participants waived written informed consent. To protect patient privacy, all imaging data were anonymized during the study, and no identifiable personal information was involved.

The inclusion criteria for patients were as follows: (1) diagnosed with Non-Small Cell Lung Cancer (NSCLC) by Yangzhong People's Hospital between January 1, 2022 and January 1, 2024; (2) underwent a standard chest CT scan before surgery, and the image quality met the requirements for subsequent imaging genomics analysis; (3) all cases were diagnosed by pathological biopsy, and the degree of differentiation was graded according to the 2021 World Health Organization (WHO) lung cancer histological classification criteria; (4) no prior anti-tumor intervention, including chemotherapy, radiotherapy, or targeted therapy.

Exclusion criteria included: (1) poor quality of preoperative CT images with severe artifacts, motion interference, or unclear lesion boundaries; (2) missing imaging data or pathology reports; (3) combined with primary malignant tumors in other locations or unclear lung cancer subtypes; (4) the size of the tumor lesion was too small (the longest diameter < 5 mm) to meet the requirements for accurate ROI delineation.

Finally, a total of 184 NSCLC patients were included, all of whom completed preoperative CT examination and pathological differentiation grade determination. According to the degree of histological pathological differentiation and the setting of the three-group binary classification strategy, the samples were distributed as follows: 27 cases in the poorly differentiated group and 157 cases in the non-poorly differentiated group; 104 cases in the moderately differentiated group and 80 cases in the non-moderately differentiated group; 53 cases in the well-differentiated group and 131 cases in the non-well-differentiated group. Figure (**1**) shows how the original three-class classification task (poorly, moderately, and well differentiated) is restructured into three independent binary classification tasks to reduce the impact of blurred class boundaries on model performance.

### 2.2 Imaging Protocol

All included patients underwent a routine chest CT examination before surgery, using scanning equipment including Philips Brilliance 64-row spiral CT (Philips Medical Systems, The Netherlands) and Siemens SOMATOM Definition Flash (Siemens Healthineers, Germany). Both devices are 64-slice spiral CTs with submillimeter spatial resolution, which can meet the requirements for fine structure display required for imaging analysis of lung tumors. During the examination, all patients were in the supine position, and deep inspiration and end-breath holding scanning were performed to reduce motion artifacts and ensure that the lung structure was clearly distinguishable. The scanning range covered the upper edge of the thorax to the lower edge of the costophrenic angle to ensure complete coverage of the lung field area and mediastinal structure. The specific scanning parameters were set as follows: tube voltage 120 kVp, automatic tube current adjustment (200–300 mA), pitch 1.0, rotation time 0.5 seconds, layer thickness 1 mm, layer spacing 1 mm, image matrix 512 × 512, and Field of View (FOV) range 350 mm.

This study did not uniformly adopt an enhanced scanning scheme. All images were collected from non-enhanced CT sequences to exclude the potential interference of contrast agents on image features and enhance the applicability and generalizability of the model. Although some patients underwent enhanced scanning, only their plain scan image sequences were extracted for subsequent analysis in this study. All images were initially quality controlled by radiologists in the Department of Medical Imaging to ensure that there were no obvious motion artifacts, truncation artifacts, or image undersampling. Image data were stored in DICOM format and exported to an independent research workstation through the in-hospital PACS system. To ensure the comparability of data across devices and patients, all images were uniformly spatially resampled (to 1 mm × 1 mm × 1 mm isotropic voxels) using the SimpleITK software library (Python 3.8 environment) in the subsequent processing stage, and the Z-score standardization method was used to complete grayscale normalization, adjusting all images to a distribution with a mean of 0 and a standard deviation of 1 to reduce the influence of the image grayscale dynamic range on the consistency of feature extraction.

### 2.3 Image Preprocessing and Tumor Segmentation

#### 2.3.1 Image Preprocessing

To eliminate the impact of different CT devices, acquisition parameters, and patient body size differences, this study performed standardized preprocessing on all DICOM format raw image data to ensure the stability and repeatability of the feature extraction process. Image preprocessing was completed by two image analysis engineers in a Python (version 3.8) environment, using SimpleITK (version 2.1.0) and the Numpy library to achieve spatial and grayscale standardization.

First, all images were resampled in three-dimensional space, and the voxel size was uniformly adjusted to 1.0 mm × 1.0 mm × 1.0 mm to ensure the consistency of image spatial resolution between different patients. Subsequently, the images were normalized using Z-score grayscale normalization, and the image grayscale values were standardized to a distribution with a mean of 0 and a standard deviation of 1. This method can effectively reduce the impact of inter-individual CT value differences on subsequent feature stability and improve the generalization ability of the model. In addition, to ensure the integrity of the lung structure, lung windows were uniformly used for image preprocessing, with the window width set to 1500 HU and the window level set to -600 HU. All processing steps were completed in unsupervised batch mode, and visual inspection was used to ensure that the preprocessing was correct.

#### 2.3.2 Tumor Segmentation

This study employed a semi-automated segmentation process, involving two senior radiologists with more than 10 years of work experience collaborating to complete the lesion ROI (Region of Interest) delineation, with the chief physician conducting the final review. All segmentation operations were performed in 3D Slicer software (version 5.2.2), using its built-in “GrowCut” and “Paint” tools for contour delineation and fine-tuning. The tumor ROI of each patient was required to completely cover the solid lesion area, avoiding the surrounding normal lung tissue, bronchi, blood vessels or cavitary structures. For patients with multiple lesions, only the largest lesion was selected for analysis. The segmentation range was based on the lung window image and was manually adjusted layer by layer and frame by frame to ensure that the contours closely fit the edge of the tumor and fully reflected the morphological characteristics of the tumor. To further control the segmentation quality and consistency, a two-person cross-review system was used to evaluate the segmentation consistency, and the lesion ROI was randomly selected from 5% of the samples for the Dice Similarity Coefficient (DSC) calculation to evaluate segmentation reproducibility. If DSC < 0.85, the case needs to be re-segmented until it meets the standard. Finally, all delineated areas were saved as .nrrd format files and spatially registered with the original CT images for subsequent radiomics feature extraction.

### 2.4 Radiomics Feature Extraction and Selection

#### 2.4.1 Radiomics Feature Extraction

In this study, PyRadiomics (version 3.0.1) was used to extract radiomic features from tumor regions in CT images. The extraction process strictly followed the feature calculation standards established by the Image Biomarker Standardisation Initiative (IBSI) to ensure that the extracted features had good repeatability and consistency across platforms and devices. Before feature extraction, all images had undergone spatial resampling and grayscale normalization, and all tumor ROIs had undergone coordinate alignment and edge smoothing to ensure the stability of subsequent feature calculations.

1,077 original image features were extracted, covering information on multiple dimensions of tumors, such as grayscale distribution, spatial morphology, and texture heterogeneity. These features are mainly derived from six categories of indicators, including shape features, first-order statistical features, Grayscale Co-occurrence Matrix (GLCM), Grayscale Run Length Matrix (GLRM), Grayscale Size Zone Matrix (GLSZM), and multi-scale wavelet transform features. Shape features are used to describe the geometric structure of the tumor. First-order statistical features reflect the overall characteristics of the grayscale distribution, texture features are used to characterize the complexity of the internal tissue structure of the tumor, and wavelet features are further revealed by filtering decomposition. Grayscale changes and edge features at multiple scales. The calculation of all features is based on the normalized image volume of isotropic voxels (1 mm^3^) to minimize the impact of individual differences in equipment and patients.

#### 2.4.2 Radiomics Feature Screening

In order to reduce the risk of overfitting caused by high-dimensional features and improve the stability and interpretability of the model, this study strictly screened the original features before modeling. First, Pearson correlation analysis was used to evaluate the linear redundancy between features. Redundant features with an absolute value of the pairwise correlation coefficient exceeding 0.90 were removed, retaining only the core variables with strong information complementarity. Subsequently, the LASSO (Least Absolute Shrinkage and Selection Operator) regression method was introduced for further screening. This method automatically converges the weights of some features to zero by introducing the L1 regularization penalty term in the loss function, thereby achieving sparse modeling and variable selection. The LASSO screening process was implemented in Scikit-learn (version 0.24.2). The optimal regularization parameter α value was determined by 10-fold cross-validation, and the solution with the smallest mean square error was selected as the final model.

### 2.5 Prediction Model Construction

Based on the extracted and screened radiomic features, this study constructed a supervised classification model for predicting the degree of histological differentiation of lung cancer. Taking into account the characteristic differences in imaging performance of different differentiation levels and the cross-ambiguity between categories, a “one-to-many” multi-task modeling strategy was adopted to establish three independent binary classification models, namely, poor differentiation *vs*. non-poor differentiation, moderate differentiation *vs*. non-moderate differentiation, and high differentiation *vs*. non-high differentiation, to enhance the model's ability to distinguish specific differentiation levels.

In this study, we employed four widely adopted ensemble learning models-Gradient Boosting Decision Tree (GBDT), Random Forest (RF), Extreme Gradient Boosting (XGBoost), and LightGBM-to construct predictive classifiers for each binary differentiation task. GBDT operates as a forward stage-wise additive model that builds decision trees sequentially by minimizing a differentiable loss function, making it effective in capturing complex nonlinear relationships in radiomic feature spaces [18]. RF utilizes a bootstrap aggregating (bagging) strategy with randomized feature selection to enhance robustness and mitigate overfitting, especially in small-sample, high-dimensional settings [19]. XGBoost improves upon traditional gradient boosting by incorporating second-order derivatives, regularization terms, and shrinkage strategies, achieving both higher accuracy and computational efficiency [20]. LightGBM, a highly scalable variant of gradient boosting, adopts a histogram-based tree growth method and leaf-wise split selection, enabling faster training speed and better performance on large datasets with high feature dimensionality [20]. These models have consistently demonstrated superior performance and stability in radiomics-based classification problems, thus serving as reliable state-of-the-art baselines for this study. All models are implemented using Python open source machine learning libraries such as Scikit-learn, XGBoost, and LightGBM (version 0.24.2, 1.5.0, and 3.3.5).

### 2.6 Model Evaluation

The division ratio of this study was 7:3 to simulate the generalization performance in real clinical scenarios. The evaluation indicators used multi-dimensional performance parameters commonly used in classification models, including accuracy, precision, recall, F1-score, and Area Under the Curve (AUC), to comprehensively reflect the performance of the model in different categories of prediction. As the core indicator for evaluating the performance of binary classification models, AUC can reflect the overall discrimination ability of the model under different classification thresholds. The closer it is to 1.0, the better the discrimination of the model. The ROC (Receiver Operating Characteristic) curve plots the relationship between the true positive rate and the false positive rate to intuitively show the trade-off between the sensitivity and specificity of the model under different classification thresholds. In order to further visualize the discrimination ability of each model, this study plotted the ROC curve for each model under each classification task, and calculated its AUC value for horizontal comparison. The overall research method is shown in Fig. (**2**).

## 3 RESULTS

### 3.1 Experimental Setup

All models have been systematically optimized for hyperparameters. Parameter tuning was performed by combining grid search (GridSearchCV) with five-fold cross-validation. Key parameters such as learning rate, maximum tree depth (max_depth), and number of estimators (n_estimators) were debugged on the training set, and the parameter combination with the best performance was selected for final modeling. In terms of data partitioning, the overall sample of the study was randomly divided into a training set and an independent test set in a ratio of 7:3, which were used for model training and generalization ability evaluation, respectively. All classification models were internally evaluated using five-fold cross-validation on the training set to reduce the volatility caused by sample partitioning; after the parameters of the final model were fixed, its performance was independently evaluated on the test set to ensure the stability and repeatability of the results. In addition, a unified random seed control was used for both the training and prediction processes to ensure the reproducibility of the experiment.

All experimental processes of this study were completed in a local Linux server environment. The operating system was Ubuntu 20.04 LTS, the development environment was based on Python 3.8.13, and the core algorithm modules were mainly implemented using the following open source libraries: SimpleITK (version 2.1.0) for image processing, PyRadiomics (version 3.0.1) for radiomics feature extraction, Scikit-learn (version 0.24.2), XGBoost (version 1.5.0), and LightGBM (version 3.3.5) for machine learning model construction and evaluation, and SHAP (version 0.41.0) for interpretability analysis. The experiment was completed on a local high-performance workstation with the following hardware configuration: Intel Xeon Gold 6226R 2.90GHz CPU × 2 (48 cores in total), 256 GB DDR4 memory, NVIDIA RTX A6000 GPU × 1 (48 GB video memory), equipped with CUDA 11.4 and cuDNN 8.2. All model training and SHAP calculations are accelerated with GPU support to improve the efficiency of feature analysis and model fitting.

### 3.2 Radiomics Feature Extraction and Selection Results

#### 3.2.1 Initial Radiomics Features

After completing image preprocessing and tumor lesion ROI segmentation, this study extracted 1077 high-throughput radiomics features in six categories through PyRadiomics, covering multi-dimensional characterization information of tumors in spatial morphology, grayscale distribution, and texture structure. Among them, 14 shape features were extracted, mainly describing the geometric structure of the tumor, such as volume, surface area, maximum diameter, *etc*.; 18 first-order statistical features were extracted to reflect the central tendency and distribution characteristics of grayscale values, such as mean, variance, kurtosis, *etc*.; texture features included GLCM (24 items), GLRLM (16 items), GLSZM (16 items), NGTDM (5 items) and GLDM (14 items), totaling 75 items, which comprehensively quantified the spatial correlation pattern, texture complexity and local heterogeneity between tumor grayscales. In order to enhance the model's ability to perceive information of different frequencies, wavelet multiscale decomposition was also applied to the image to generate 8 sets of frequency domain images (such as HHL, LLH, LHH, *etc*.), and first-order and texture features were repeatedly extracted for each set of images. After wavelet enhancement, a total of 864 transform domain features were extracted, which greatly improved the sensitivity to details, edges, and texture microstructures. Overall, the original imaging omics features are high-dimensional and highly redundant, and need to be further screened to avoid overfitting and dimensionality disaster.

#### 3.2.2 Feature Selection and LASSO Visualization

This study uses the LASSO (Least Absolute Shrinkage and Selection Operator) method to reduce the dimension of the initial features. LASSO achieves feature selection while compressing the regression coefficients by introducing the L1 regularization penalty term. To ensure the robustness of the screening results, a 10-fold cross-validation method is used with the Mean Squared Error (MSE) as the evaluation index to determine the optimal regularization parameter α. Figure (**3A**) shows the change trajectory of each feature coefficient as the regularization strength (α value) gradually increases in the three groups of classification tasks (LASSO path diagram). It can be observed that most features remain non-zero when the α value is small, and gradually become sparse as the penalty increases, and only a few key features are retained in the final model. Figure (**3B**) shows the corresponding LASSO regression mean square error trend curve (with standard deviation) as α changes. It can be seen that the MSE reaches its lowest point and stabilizes near a certain inflection point, indicating that this is the position of the optimal regularization strength.

### 3.3 Machine Learning Model Results

This study evaluated the prediction performance of four ensemble learning models (GBDT, RF, XGB, and LGBM) on three independent binary classification tasks (poorly differentiated *vs*. non-poorly differentiated, moderately differentiated *vs*. non-moderately differentiated, and highly differentiated *vs*. non-highly differentiated). The performance evaluation indicators include accuracy, precision, recall, F1-score, and Area Under the Curve (AUC). The results are shown in Table **1**, and the ROC curves are shown in Figs. (**4**-**6**).

In the poorly differentiated *vs*. non-poorly differentiated task, XGBoost achieved the best overall accuracy (ACC = 0.8393) and an F1-score of 0.6879. However, its AUC was only 0.7005, indicating that although the model has strong overall classification accuracy, its category discrimination ability is still limited. In contrast, the GBDT model has the highest AUC (0.849), indicating that it has better discrimination ability in distinguishing poorly differentiated from non-poorly differentiated patients, while maintaining good recall (0.7292) and an F1-score (0.6711), which has potential advantages in reducing missed diagnoses. The RF model performed slightly worse, especially in terms of precision and AUC, while the LGBM model was at a relative disadvantage in all indicators of this task, suggesting that it is not stable enough in the poorly differentiated classification scenario.

In the moderately differentiated *vs*. non-moderately differentiated task, the performance of all models was low overall, with AUCs not exceeding 0.55. The AUCs of GBDT and LGBM were 0.5456 and 0.5482, respectively, indicating that the imaging phenotypes of moderately differentiated patients were highly heterogeneous and had fuzzy boundaries, making it difficult for the model to establish effective distinctions. Although the recall of LGBM reached 0.5677, indicating high sensitivity, the precision was only 0.5667, with a high false positive rate. The performance of the XGB and RF models was similar, both at a low level, indicating that there was still room for optimization of the existing imaging genomics features and classification methods in the moderately differentiated identification task.

In the well-differentiated *vs*. non-well-differentiated task, the RF model achieved the highest AUC value (0.7188), outperforming GBDT (0.7016) and XGB (0.6984), showing its strong generalization ability in identifying well-differentiated tumors. Although the four models are similar in accuracy (all about 0.66), there are slight differences in precision and F1-score. GBDT has the highest F1-score (0.6166), indicating that it has achieved a good balance between “reducing misjudgment” and “enhancing recall.” LGBM performed stably in this task with an AUC of 0.6781, but was slightly inferior to RF and GBDT.

Figures (**4**-**6**) further shows the ROC curves of each model in different classification tasks. It can be observed that in the low differentiation task, the ROC curve of GBDT is better than other models as a whole, with the largest product under the curve, which verifies its excellent discrimination ability; while in the high differentiation task, the RF curve performs best, especially in the low false positive rate area, with a high TPR, which has good clinical screening potential. The ROC curves of the medium differentiation task are generally close to the diagonal line, indicating that the classification of this task is difficult. In addition, the confusion matrices of all models are shown in Supplementary Material **1**.

### 3.4 Model Interpretability Analysis

This study uses the SHAP (Shapley Additive Explanations) method to analyze the global feature importance of the GBDT model with the best performance. The SHAP summary graphs of the GBDT model for the three binary classification tasks-low differentiation, moderate differentiation, and high differentiation-are shown, respectively. Each horizontal line in the figure represents an imaging feature; the color of the point reflects the size of the feature value (red is high, blue is low), and the horizontal distribution indicates the degree and direction of the corresponding feature's influence on the model output (SHAP value). The ranking on the right side of the figure is the feature importance.

**Fig. (7):**
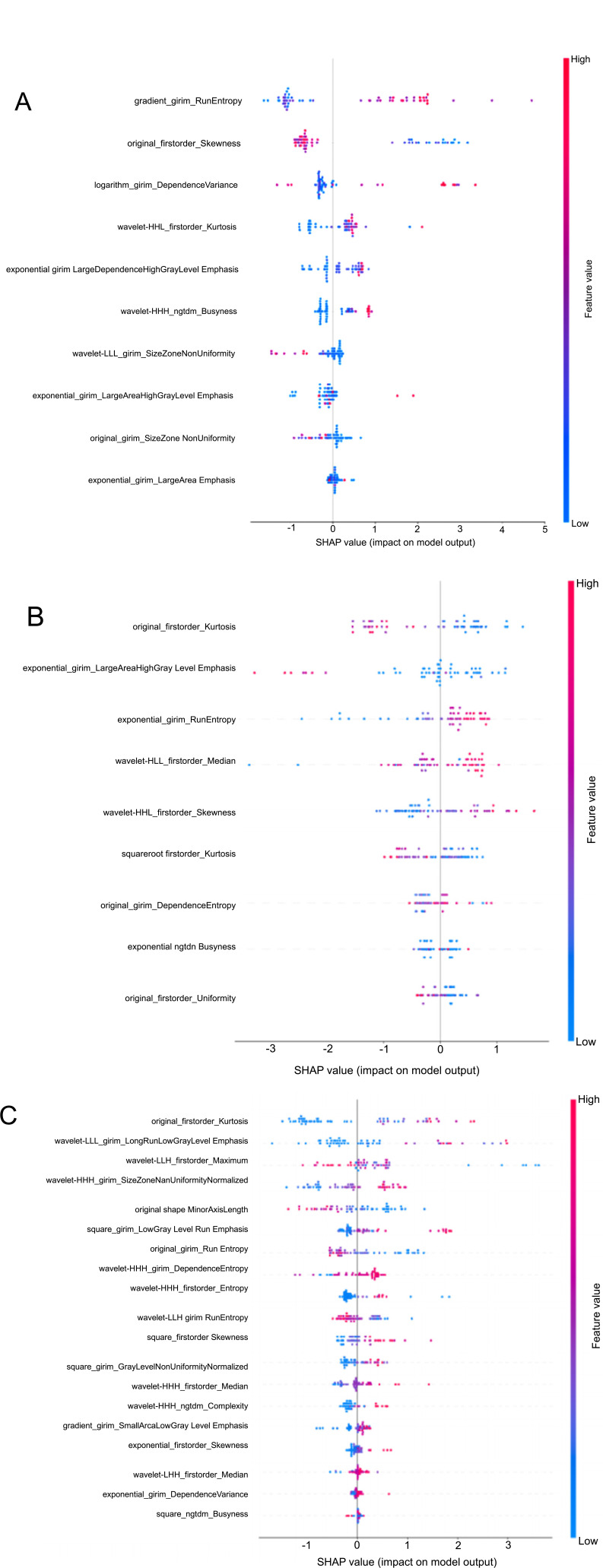
SHAP analysis results of each model. **A**-**C** represent the results of low differentiation, moderate differentiation, and high differentiation groups, respectively.

In the poorly differentiated *vs*. non-poorly differentiated task (Fig. **7A**), the top-ranked key feature is gradient_ glrlm_RunEntropy, indicating that the run entropy of tumor texture has a strong discriminative power in predicting poorly differentiated status. This feature describes the complexity of the grayscale value repetitive pattern. The higher the entropy value, the more disordered the tumor tissue structure, which may be related to the disordered arrangement of poorly differentiated tumor cells. Secondly, original_firstorder_ Skewness and logarithm_gldm_DependenceVariance also showed high SHAP values, providing complementary information from the two dimensions of grayscale skewness and grayscale dependency heterogeneity. In addition, a series of wavelet domain features (such as wavelet-HHL_firstorder_Kurtosis and wavelet-HHH_ngtdm_Busyness) also showed strong contributions, indicating that high-frequency texture components have potential value in identifying highly heterogeneous tumors.

In the moderately differentiated *vs*. non-moderately differentiated task (Fig. **7B**), the order of feature importance changed. original_firstorder_Kurtosis became the most contributing feature, suggesting that the kurtosis of the pixel distribution in the image plays a dominant role in the identification of moderately differentiated tissue. In general, higher kurtosis indicates more extreme grayscale values in the image, which may reflect the obvious local density differences in tumor tissue. In addition, exponential_glszm_ LargeAreaHighGrayLevelEmphasis and exponential_glrlm_ RunEntropy indicate that grayscale concentration and texture complexity still play a key role in model discrimination. Wavelet features such as wavelet-HLL_firstorder_Median also rank at the top, suggesting that the local median is of certain significance for identifying moderately differentiated lesions. Overall, the distribution of feature importance of the moderately differentiated model is relatively scattered, which may be related to the large biological heterogeneity and blurred boundaries of this category itself.

In the well-differentiated *vs*. non-well-differentiated tasks (Fig. **7C**), original_firstorder_Kurtosis ranked first again, indicating that this statistical feature has a stable contribution across differentiation levels. Compared with the poorly differentiated tasks, the well-differentiated model relies more on features with lower original spatial and texture complexity, such as wavelet-LLH_firstorder_Maximum and original_ shape_MinorAxisLength, reflecting that its image performance is more regular and consistent. In addition, texture uniformity indicators such as glrlm_LongRunLowGrayLevelEmphasis and glszm_SizeZoneNonUniformityNormalized rank high, further indicating that well-differentiated tumors are more homogeneous in texture patterns and the model is easier to identify.

In summary, SHAP analysis revealed the differences in the role of radiomics features corresponding to different differentiation levels in the model. Texture entropy and grayscale skewness dominated the low differentiation prediction task. The medium differentiation task showed mixed and unstable multi-source features, and the high differentiation prediction mainly relied on shape, grayscale uniformity and, low-frequency texture features.

## 4 DISCUSSION

This study focuses on the problem of accurate prediction of the degree of preoperative histological differentiation of lung cancer and constructs a multi-task modeling framework based on CT radiomics and machine learning algorithms. Unlike the previous problem setting of dividing the differentiation level into a single three-class classification, we innovatively decomposed the prediction task into three independent binary classification models. These models identify poorly differentiated, moderately differentiated, and well-differentiated tumors, respectively, to reduce the impact of feature overlap between categories on model performance. In terms of feature engineering, this study systematically extracted 1077-dimensional multi-type radiomics features, performed sparse screening through the LASSO method, and finally constructed a feature subset with high discriminability and low redundancy. In model construction, we comprehensively compared integrated learning algorithms such as GBDT, RF, XGB, and LGBM. The results showed that GBDT performed best in predicting poor differentiation, while RF had better generalization ability in the task of high differentiation. In addition, combined with SHAP interpretability analysis, this study further revealed the key imaging phenotypic features corresponding to different differentiation levels, providing a clear explanation path for model output and data support for future biomarker exploration. Overall, the method proposed in this study not only ensures performance but also takes into account clinical interpretability, providing a new strategy for preoperative non-invasive pathological classification of lung cancer patients.

This research method considers the feasibility and clinical needs in designing the technical route, resulting in an overall solution and good practicality, and promotion potential. First, in terms of model selection, we introduced a variety of ensemble learning algorithms that are currently widely used in medical image analysis tasks, including GBDT, RF, XGB, and LGBM. These models have strong fitting capabilities for high-dimensional features, and can still maintain good robustness when the sample size is relatively limited. They are particularly suitable for the application background of “few samples - high dimensions” problems in imaging omics scenarios. The experimental results show that GBDT and RF achieve the best performance in low-differentiation and high-differentiation tasks, respectively, verifying their characteristics of stability and flexibility in medical classification problems. Compared with traditional logistic regression or single neural network structures, the integrated algorithm not only performs better in performance, but also facilitates seamless docking with interpretable analysis (such as SHAP) to enhance the transparency of the model. Secondly, decomposing the traditional three-classification task into three independent two-classification models is an important strategic innovation of this study. This design effectively avoids the problem of category confusion caused by blurred boundaries and significant feature overlap among the three types of tumor differentiation levels. By treating each differentiation level as an independent “positive class” and merging it with other levels to form a “negative class,” the model can focus more on learning the features of each type of tumor, thereby improving the discrimination accuracy and generalization ability of a single task. Especially in the case of unbalanced sample size, this strategy helps alleviate the common “minority class being ignored” problem in multi-classification tasks. In addition, the independence of the three tasks allows each group of models to be differentially tuned to participate in feature optimization according to the specific task characteristics, enhancing the flexibility of the overall framework.

Based on radiomics and machine learning, this study established a non-invasive strategy for the preoperative prediction of the degree of histological differentiation of lung cancer, which has clear clinical translational value. At present, the differentiation assessment of lung cancer still mainly relies on pathological analysis of tissue samples obtained after surgery or puncture. Although the accuracy is high, there are certain limitations, such as the strong trauma of the operation, limited sample acquisition, and reliance on the experience of the pathologist [21-23]. In particular, in clinical situations where puncture is not suitable, the sample is insufficient, or the tumor is highly heterogeneous, traditional pathological methods often cannot provide comprehensive and stable differentiation information. The prediction model based on CT radiomics proposed in this study can provide auxiliary evaluation of the differentiation status of lung cancer before surgery without additional intervention measures, and has good accessibility and practicality. From the perspective of treatment decision-making, the degree of differentiation is an important factor affecting the selection of individualized treatment plans for lung cancer patients. Poorly differentiated tumors usually indicate more aggressive biological behavior and may be more suitable for extended surgery, preoperative neoadjuvant therapy, or intensive follow-up; while well-differentiated tumors often progress slowly, and some patients may consider conservative strategies or function-first treatment methods [24]. Therefore, accurate knowledge of the differentiation level before surgery can help doctors be more forward-looking and individualized when formulating treatment plans, thereby improving treatment effectiveness. In addition, combined with SHAP analysis, this study also provides a visual explanation of key imaging features, which helps clinicians understand the basis of model predictions, enhance trust, and promote the practical application of artificial intelligence tools in the front line of imaging diagnosis and treatment.

## 5 STUDY LIMITATIONS

Although this study has achieved relatively positive results in method design and experimental validation, there are still some limitations that need to be further optimized and improved in subsequent studies. First, the data comes from a single-center retrospective cohort with a relatively limited sample size and uneven distribution of samples across different differentiation levels, which may affect the generalization ability and stability of the model. In the task of intermediate differentiation, the model's overall performance is low, indicating that the boundary fuzziness of this type of imaging phenotype is strong and the existing feature expression ability still needs improvement. In addition, since some patients only provide non-enhanced CT images, the microvascular distribution or functional imaging features inside the tumor may not be captured, which limits the model's deep characterization of biological heterogeneity. Secondly, although this study used methods such as LASSO and SHAP to enhance the interpretability of the model, the problem of bridging deep features and biological significance has not been completely solved. The currently extracted imaging omics features mainly rely on the traditional feature dimensions defined manually, lacking comprehensive mining of potential high-level semantic information. In the future, it is possible to consider introducing deep learning automatic feature extraction or multimodal fusion strategies (such as PET/CT, clinical indicators) to further enhance the expressiveness and interpretation ability of the model. Finally, although this study technically verified the independence and effectiveness of the three binary classification models, it has not yet been deployed in real clinical processes to verify their operability and physician acceptance. In practical applications, the model output needs to be combined with other diagnostic information for multi-dimensional decision-making. Therefore, prospective validation should be conducted in a multi-center, real-world environment in the future, and the interactive interface and decision logic should be optimized in combination with physician feedback.

## CONCLUSION

This study constructed a non-invasive model based on radiomics features and integrated machine learning algorithms for preoperative prediction of the histological differentiation of non-small cell lung cancer. By innovatively using three independent binary classification tasks to replace the traditional three-classification strategy, the discrimination ability and stability of the model in identifying differentiation at all levels were significantly improved. In the model performance evaluation, GBDT and RF demonstrated excellent classification capabilities across different tasks, and the explanatory analysis combined with the SHAP method further revealed several stable and reproducible key imaging features. This strategy not only enhances the clinical interpretability of the model but also provides a preoperative auxiliary evaluation tool with potential promotional benefits for lung cancer patients. In the future, combining multimodal data fusion with multicenter verification is expected to further enhance the model's clinical applicability and accelerate the translational application of artificial intelligence technology in the precision diagnosis and treatment of lung cancer.

## Figures and Tables

**Fig. (1):**
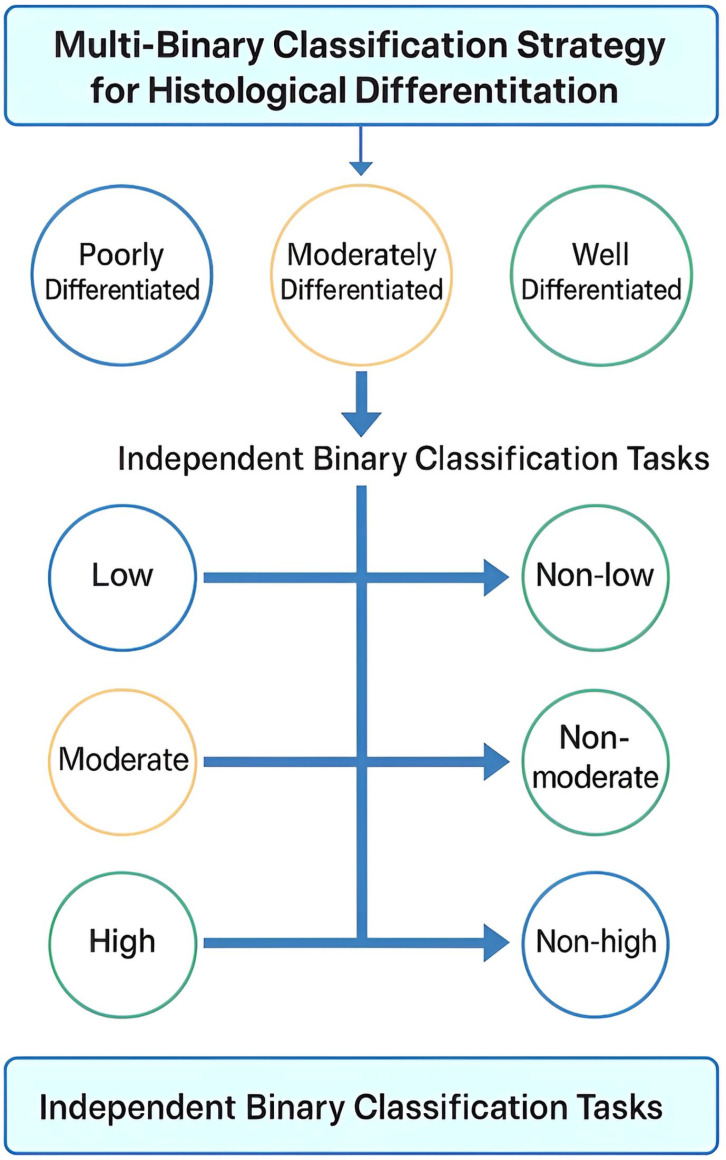
Multi-binary classification strategy for histological differentiation.

**Fig. (2):**
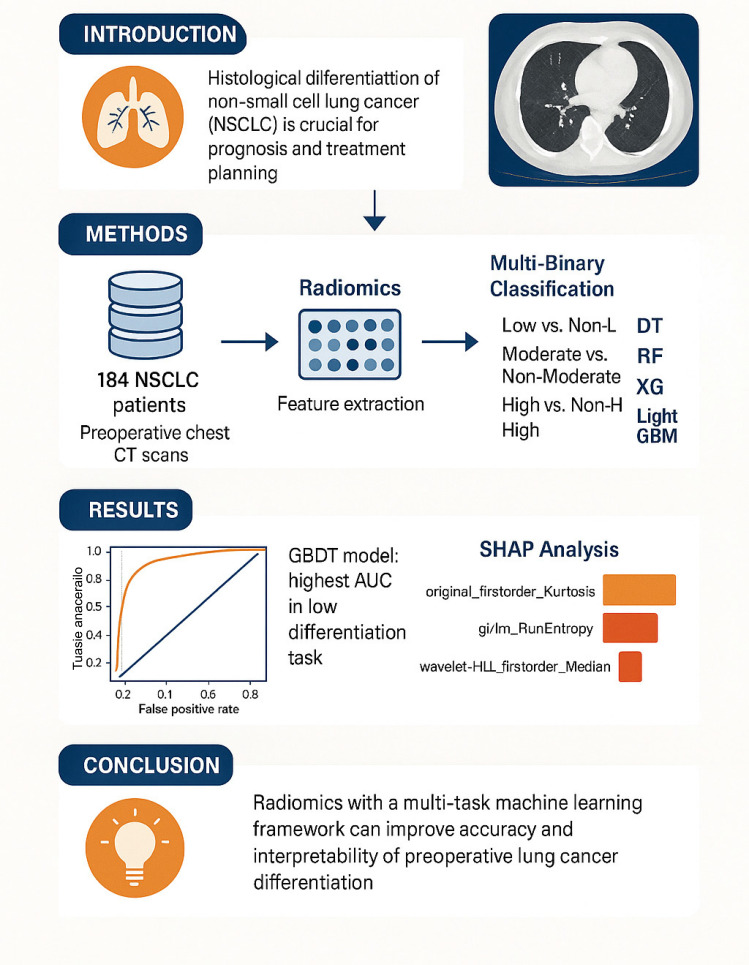
Radiomics-based modeling workflow for NSCLC differentiation.

**Fig. (3):**
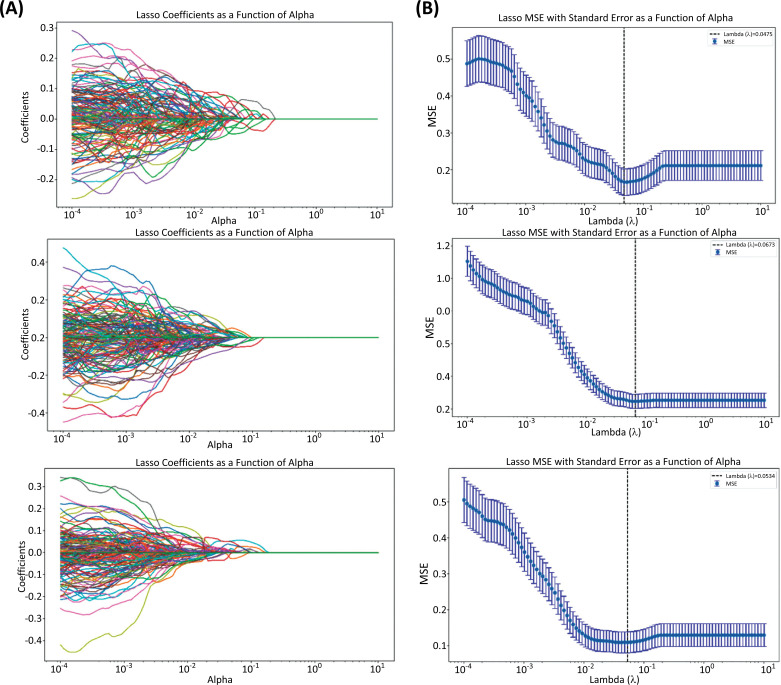
Lasso screening visualization. **A**) shows the change trajectory of each feature coefficient (LASSO path diagram) in the three classification tasks as the regularization strength (α value) gradually increases; **B**) shows the corresponding trend curve of the LASSO regression mean square error with the change of α (with standard deviation). From top to bottom are the low differentiation group, the medium differentiation group, and the high differentiation group.

**Fig. (4):**
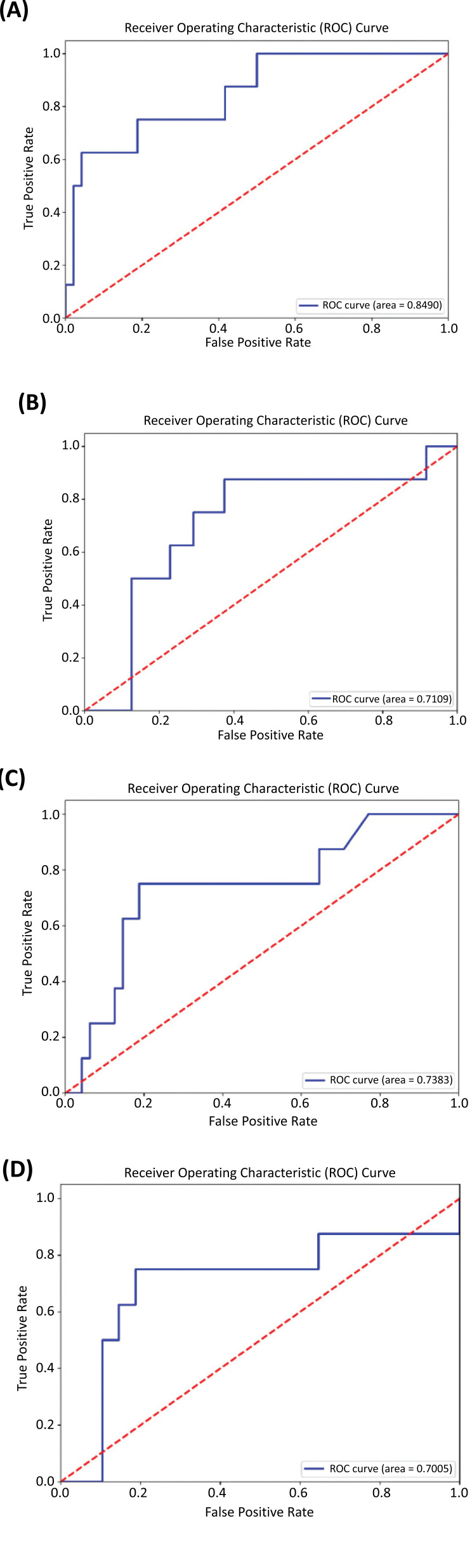
ROC curve and AUC value of the poorly differentiated group. **A**-**D** are the results of GBDT, LGBM, RF, and XGB, respectively.

**Fig. (5):**
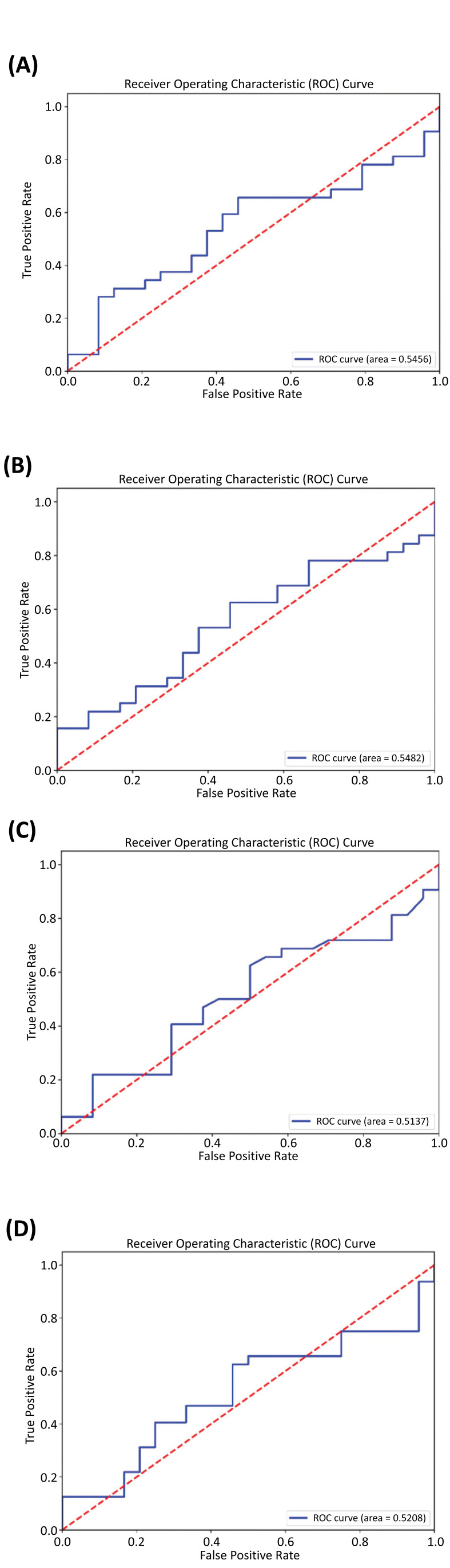
ROC curve and AUC value of the medium-differentiated group. **A**-**D** are the results of GBDT, LGBM, RF, and XGB, respectively.

**Fig. (6):**
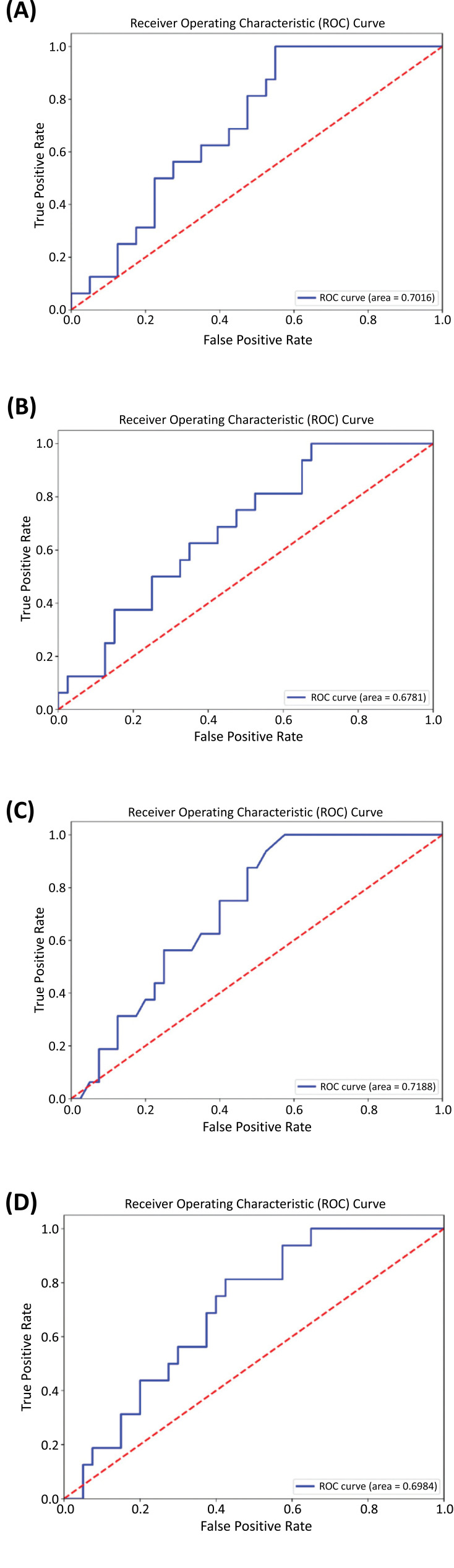
ROC curve and AUC value of the well-differentiated group. **A**-**D** are the results of GBDT, LGBM, RF, and XGB, respectively.

**Table 1:** Evaluation indicators of each model in each group.

**Group**	**Model**	**Acc**	**Precision**	**Recall**	**F1-score**	**AUC**
Low differentiation	GBDT	0.8036	0.6574	0.7292	0.6777	0.849
	RF	0.8036	0.6374	0.6771	0.6514	0.7383
	XGB	0.8393	0.6797	0.6979	0.6879	0.7005
	LGBM	0.7321	0.5846	0.6354	0.5896	0.7109
Medium differentiation	GBDT	0.5714	0.5769	0.5781	0.5709	0.5456
	RF	0.5179	0.5204	0.5208	0.5165	0.5137
	XGB	0.5357	0.5469	0.5469	0.5357	0.5208
	LGBM	0.5714	0.5667	0.5677	0.5665	0.5482
High differentiation	GBDT	0.6607	0.6143	0.6312	0.6166	0.7016
	RF	0.6607	0.5905	0.5905	0.5919	0.7188
	XGB	0.6607	0.5905	0.5938	0.5919	0.6984
	LGBM	0.6607	0.6024	0.6125	0.6052	0.6781

## Data Availability

The datasets used or analyzed during the current study are available from the corresponding author [Y.H] on reasonable request.

## LIST OF ABBREVIATIONS

NSCLC: Non-Small Cell Lung Cancer
SHAP: Shapley Additive Explanations
CT: Computed Tomography
WHO: World Health Organization
DSC: Dice Similarity Coefficient
GLCM: Grayscale Co-occurrence Matrix
GLRM: Grayscale Run Length Matrix
GLSZM: Grayscale Size Zone Matrix
LASSO: Least Absolute Shrinkage and Selection Operator
GBDT: Gradient Boosting Decision Tree
RF: Random Forest
XGBoost: Extreme Gradient Boosting
AUC: Area Under the Curve
ROC: Receiver Operating Characteristic

## STANDARDS OF REPORTING

STROBE guidelines were followed.

## References

[1] Lee E., Kazerooni E.A. (2022). Lung cancer screening.. Semin Respir Crit Care Med.

[2] Bade B.C., Dela Cruz C.S. (2020). Lung cancer 2020.. Clin Chest Med.

[3] Nasim F., Sabath B.F., Eapen G.A. (2019). Lung cancer.. Med Clin North Am.

[4] Abu Rous F., Singhi E.K., Sridhar A., Faisal M.S., Desai A. (2023). Lung cancer treatment advances in 2022.. Cancer Invest.

[5] Kaiser A.M., Gatto A., Hanson K.J., Zhao R.L., Raj N., Ozawa M.G., Seoane J.A., Bieging-Rolett K.T., Wang M., Li I., Trope W.L., Liou D.Z., Shrager J.B., Plevritis S.K., Newman A.M., Van Rechem C., Attardi L.D. (2023). p53 governs an AT1 differentiation programme in lung cancer suppression.. Nature.

[6] Tian Y., Xu L., Li X., Li H., Zhao M. (2023). SMARCA4: Current status and future perspectives in non-small-cell lung cancer.. Cancer Lett.

[7] Li Z, Zhou B, Zhu X (2023). Differentiation-related genes in tumor-associated macrophages as potential prognostic biomarkers in non-small cell lung cancer.. Front Immunol.

[8] Ingels J., De Cock L., Stevens D., Mayer R.L., Théry F., Sanchez G.S., Vermijlen D., Weening K., De Smet S., Lootens N., Brusseel M., Verstraete T., Buyle J., Van Houtte E., Devreker P., Heyns K., De Munter S., Van Lint S., Goetgeluk G., Bonte S., Billiet L., Pille M., Jansen H., Pascal E., Deseins L., Vantomme L., Verdonckt M., Roelandt R., Eekhout T., Vandamme N., Leclercq G., Taghon T., Kerre T., Vanommeslaeghe F., Dhondt A., Ferdinande L., Van Dorpe J., Desender L., De Ryck F., Vermassen F., Surmont V., Impens F., Menten B., Vermaelen K., Vandekerckhove B. (2024). Neoantigen-targeted dendritic cell vaccination in lung cancer patients induces long-lived T cells exhibiting the full differentiation spectrum.. Cell Rep Med.

[9] Chen M., Copley S.J., Viola P., Lu H., Aboagye E.O. (2023). Radiomics and artificial intelligence for precision medicine in lung cancer treatment.. Semin Cancer Biol.

[10] Wu G., Jochems A., Refaee T., Ibrahim A., Yan C., Sanduleanu S., Woodruff H.C., Lambin P. (2021). Structural and functional radiomics for lung cancer.. Eur J Nucl Med Mol Imaging.

[11] Avanzo M., Stancanello J., Pirrone G., Sartor G. (2020). Radiomics and deep learning in lung cancer.. Strahlenther Onkol.

[12] Tunali I, Gillies RJ, Schabath MB (2021). Application of radiomics and artificial intelligence for lung cancer precision medicine.. Cold Spring Harb Perspect Med.

[13] Chen X., Fang M., Dong D., Wei X., Liu L., Xu X., Jiang X., Tian J., Liu Z. (2018). A radiomics signature in preoperative predicting degree of tumor differentiation in patients with non–small cell lung cancer.. Acad Radiol.

[14] Najjar R. (2023). Redefining radiology: A review of artificial intelligence integration in medical imaging.. Diagnostics.

[15] Ghosh D, Mastej E, Jain R, Choi YS (2022). Causal inference in radiomics: Framework, mechanisms, and algorithms.. Front Neurosci.

[16] Ye J.Y., Fang P., Peng Z.P., Huang X.T., Xie J.Z., Yin X.Y. (2023). A radiomics-based interpretable model to predict the pathological grade of pancreatic neuroendocrine tumors.. Eur Radiol.

[17] Liu Z, Luo C, Chen X (2024). Noninvasive prediction of perineural invasion in intrahepatic cholangiocarcinoma by clinicoradiological features and computed tomography radiomics based on interpretable machine learning: A multicenter cohort study.. Int J Surg.

[18] Cai WY, Guo JH, Zhang MY, Ruan ZX, Zheng XC, Lv SS (2020). GBDT-based fall detection with comprehensive data from posture sensor and human skeleton extraction.. J Healthc Eng.

[19] Hu J., Szymczak S. (2023). A review on longitudinal data analysis with random forest.. Brief Bioinform.

[20] Herrera-Camacho J., Tırınk C., Parra-Cortés R.I., Bayyurt L., Uskenov R., Omarova K., Makhanbetova A., Chekirov K., Chay-Canul A.J. (2025). Body weight estimation in Holstein × Zebu Crossbred Heifers: Comparative analysis of XGBoost and LightGBM Algorithms.. Vet Med Sci.

[21] Moreira A.L., Ocampo P.S.S., Xia Y., Zhong H., Russell P.A., Minami Y., Cooper W.A., Yoshida A., Bubendorf L., Papotti M., Pelosi G., Lopez-Rios F., Kunitoki K., Ferrari-Light D., Sholl L.M., Beasley M.B., Borczuk A., Botling J., Brambilla E., Chen G., Chou T.Y., Chung J.H., Dacic S., Jain D., Hirsch F.R., Hwang D., Lantuejoul S., Lin D., Longshore J.W., Motoi N., Noguchi M., Poleri C., Rekhtman N., Tsao M.S., Thunnissen E., Travis W.D., Yatabe Y., Roden A.C., Daigneault J.B., Wistuba I.I., Kerr K.M., Pass H., Nicholson A.G., Mino-Kenudson M. (2020). A grading system for invasive pulmonary adenocarcinoma: A proposal from the international association for the study of lung cancer pathology committee.. J Thorac Oncol.

[22] Nafees B., Lloyd A.J., Dewilde S., Rajan N., Lorenzo M. (2017). Health state utilities in non–small cell lung cancer: An international study.. Asia Pac J Clin Oncol.

[23] Fujikawa R., Muraoka Y., Kashima J., Yoshida Y., Ito K., Watanabe H., Kusumoto M., Watanabe S., Yatabe Y. (2022). Clinicopathologic and genotypic features of lung adenocarcinoma characterized by the international association for the study of lung cancer grading system.. J Thorac Oncol.

[24] Hendifar A.E., Marchevsky A.M., Tuli R. (2017). Neuroendocrine tumors of the lung: Current challenges and advances in the diagnosis and management of well-differentiated disease.. J Thorac Oncol.

