# Supplementary material for: Non-invasive Prediction of Lung Cancer Histological Differentiation via Radiomics and Multi-binary Classification Models
Source: Curr Med Imaging. 2025 Oct 27;22:e15734056415019. doi: 10.2174/0115734056415019251016111351 (PMC13463227; doi:10.2174/0115734056415019251016111351)
Supplement: Supplementary file 1 [file CMIM-22-E15734056415019_SD1.pdf]

1875-6603/26 *Send Orders for Reprints to reprints@benthamscience.net*

BENTHAM SCIENCE

CrossMark

Current Medical Imaging

Content list available at: https://benthamscience.com/journals/cmir

Supplementary Material

Non-invasive Prediction of Lung Cancer Histological Differentiation *via* Radiomics and Multi-binary Classification Models

Haoran Jiang[1,#], Beichuan Zhu[2,#], Lin Xia[3] and Yingying Han[1,*]

[1]*School of Health Science and Engineering, University of Shanghai for Science and Technology, Shanghai, China*
[2]*Department of Medical Affairs, People's Hospital of Yangzhong City, Yangzhong, Jiangsu, China*
[3]*Department of Pathology, People's Hospital of Yangzhong City, Yangzhong, Jiangsu, China*

High *vs.* Non High

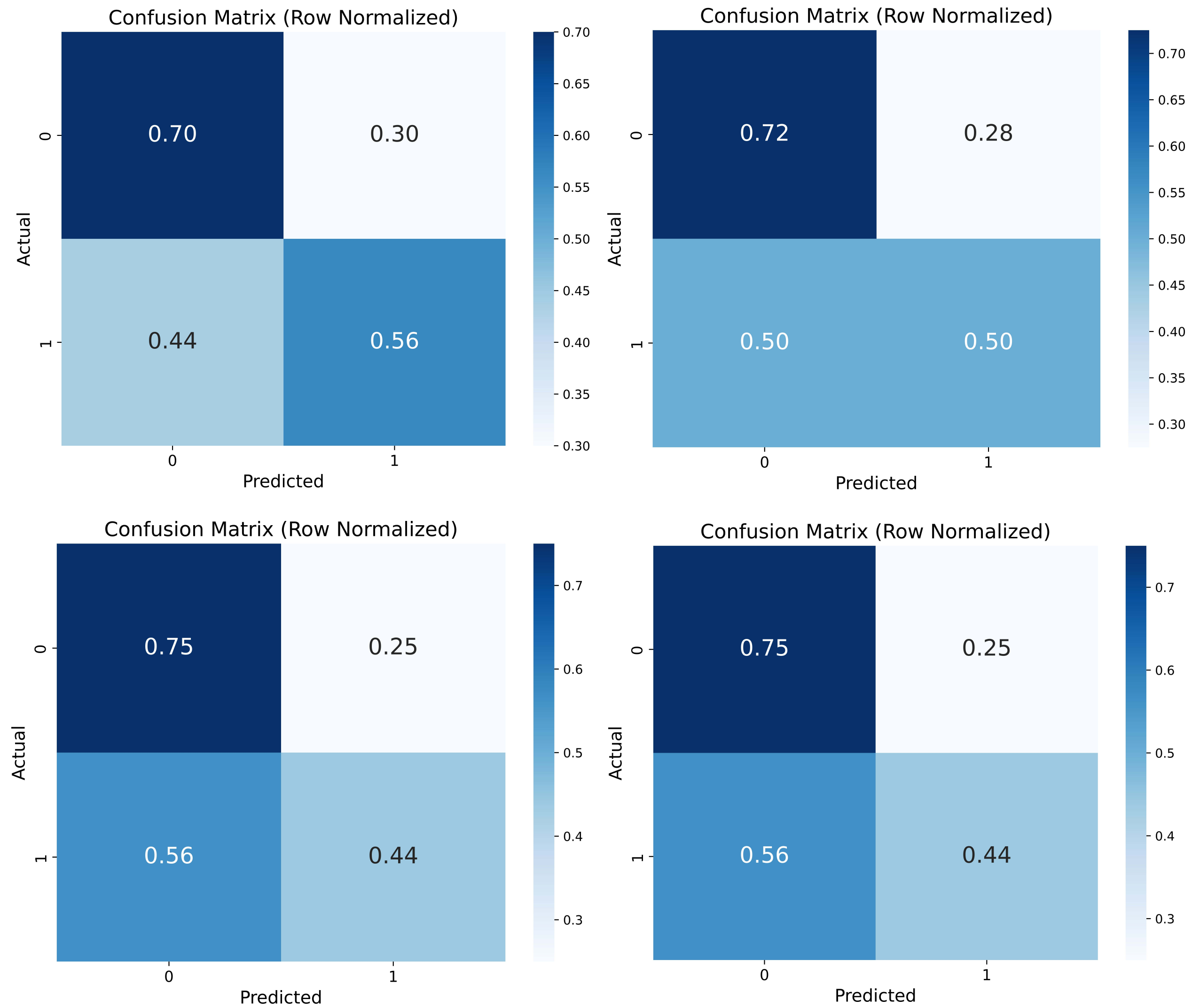

DOI: 10.2174/0115734056415019251016111351, 2026, *22*, e15734056415019

Low *vs.* Non Low

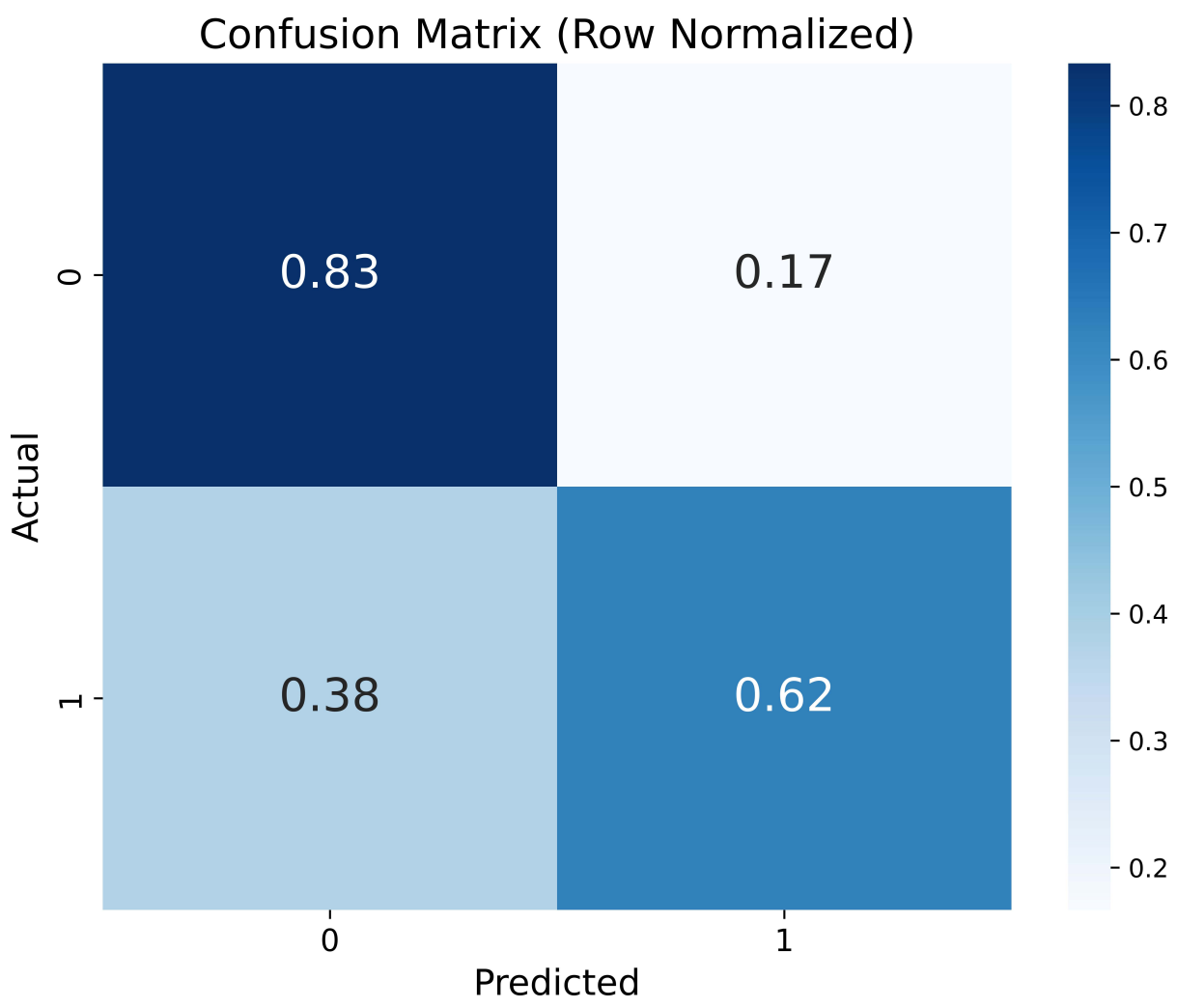

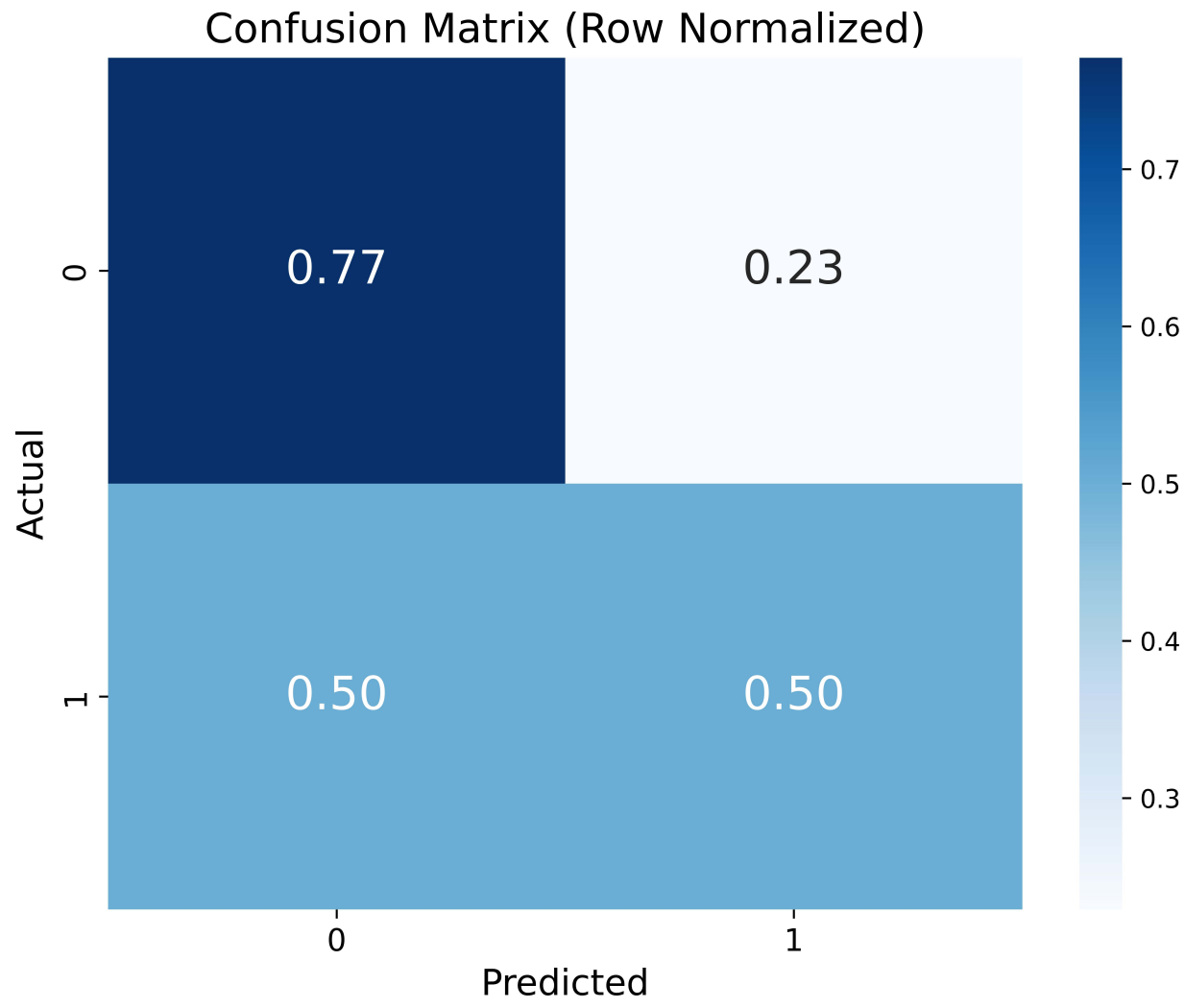

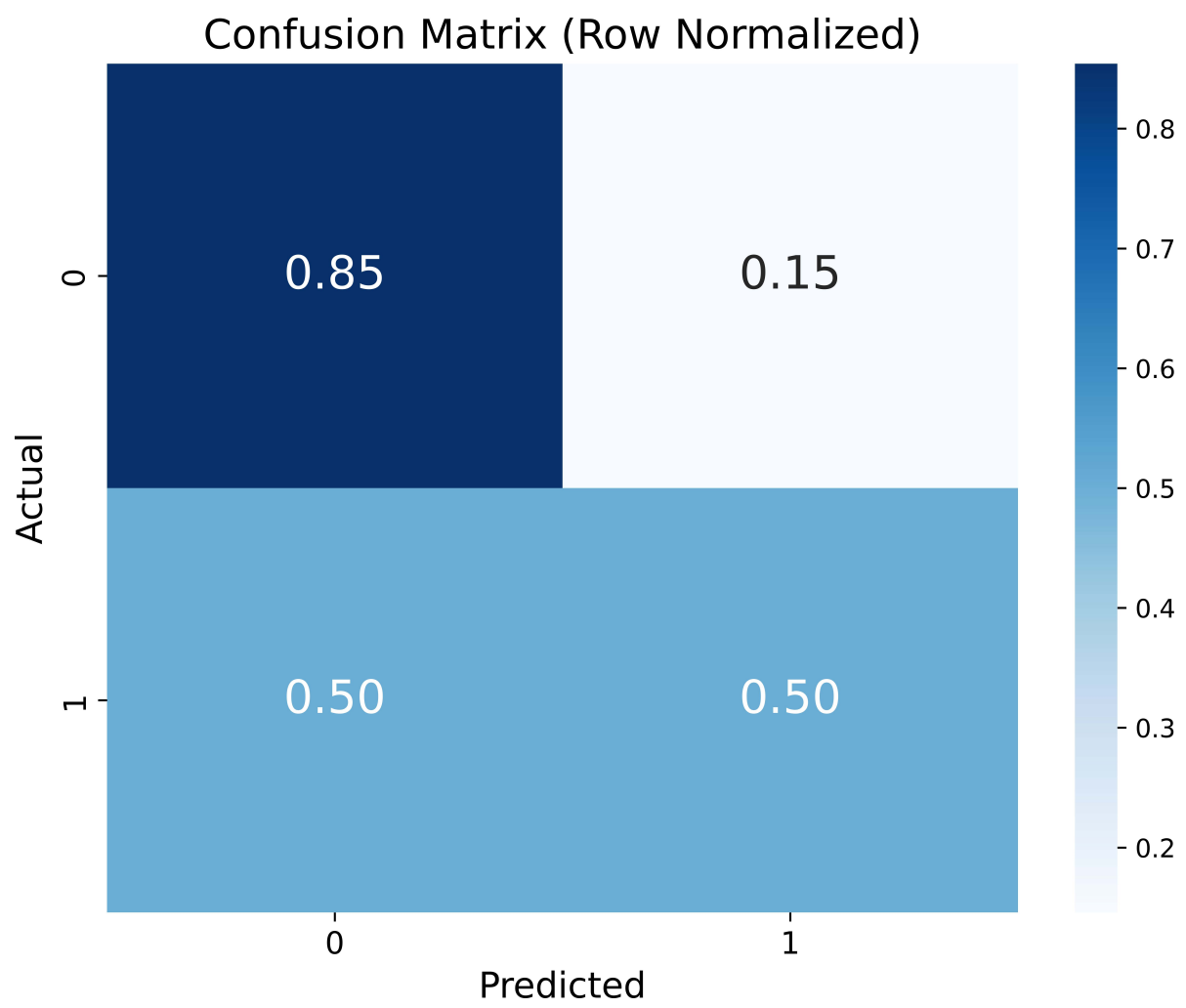

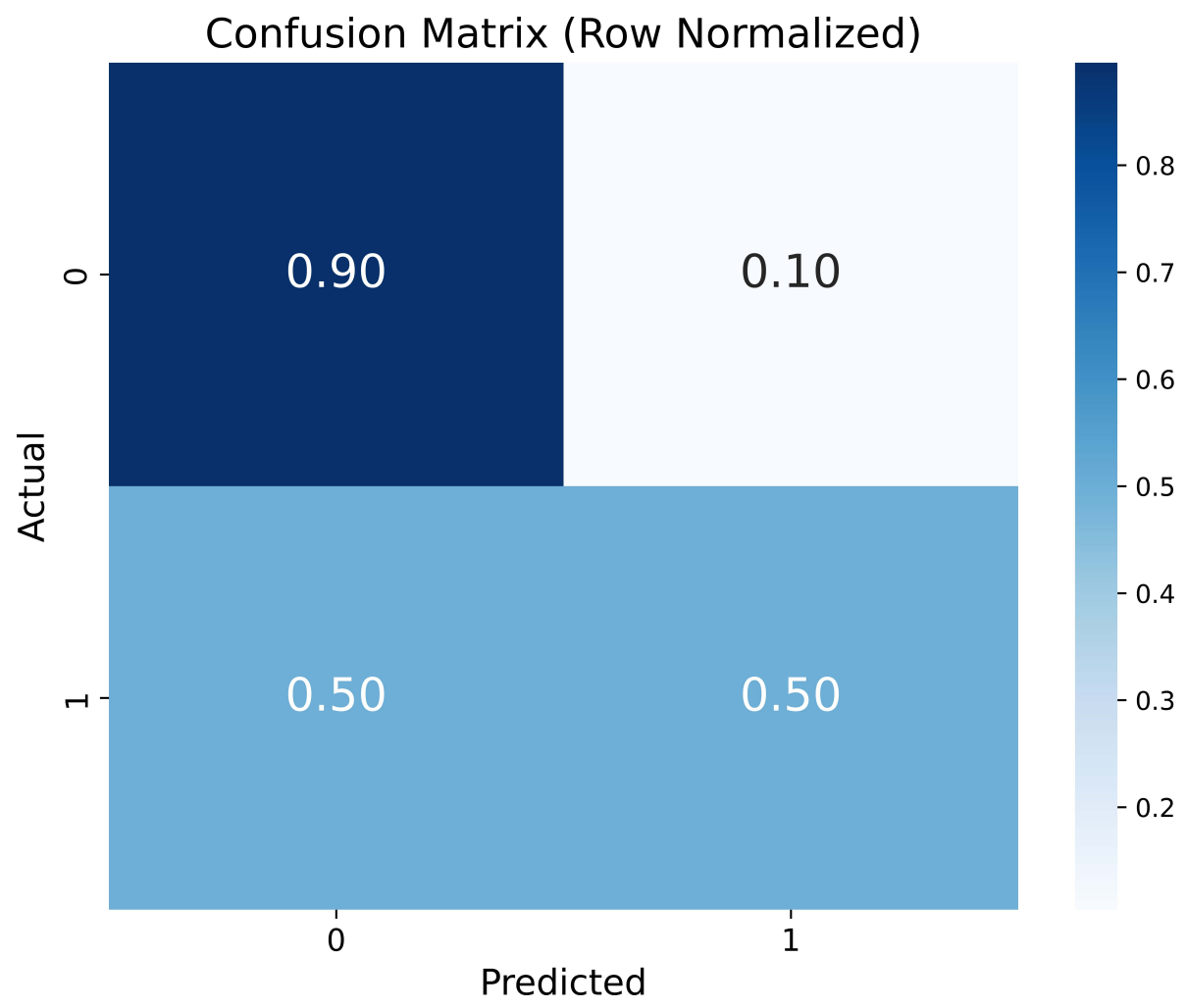

Median *vs.* Non Median

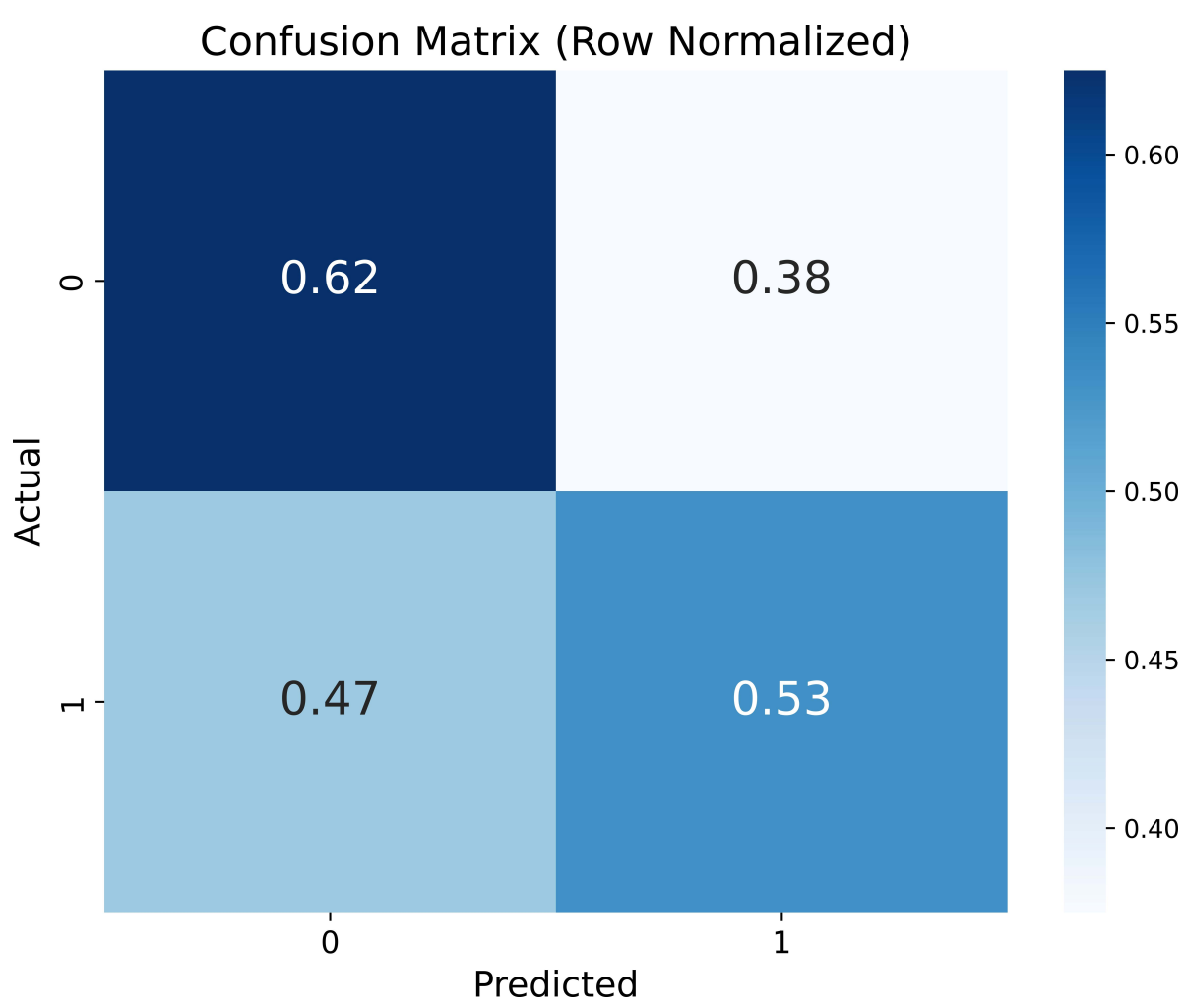

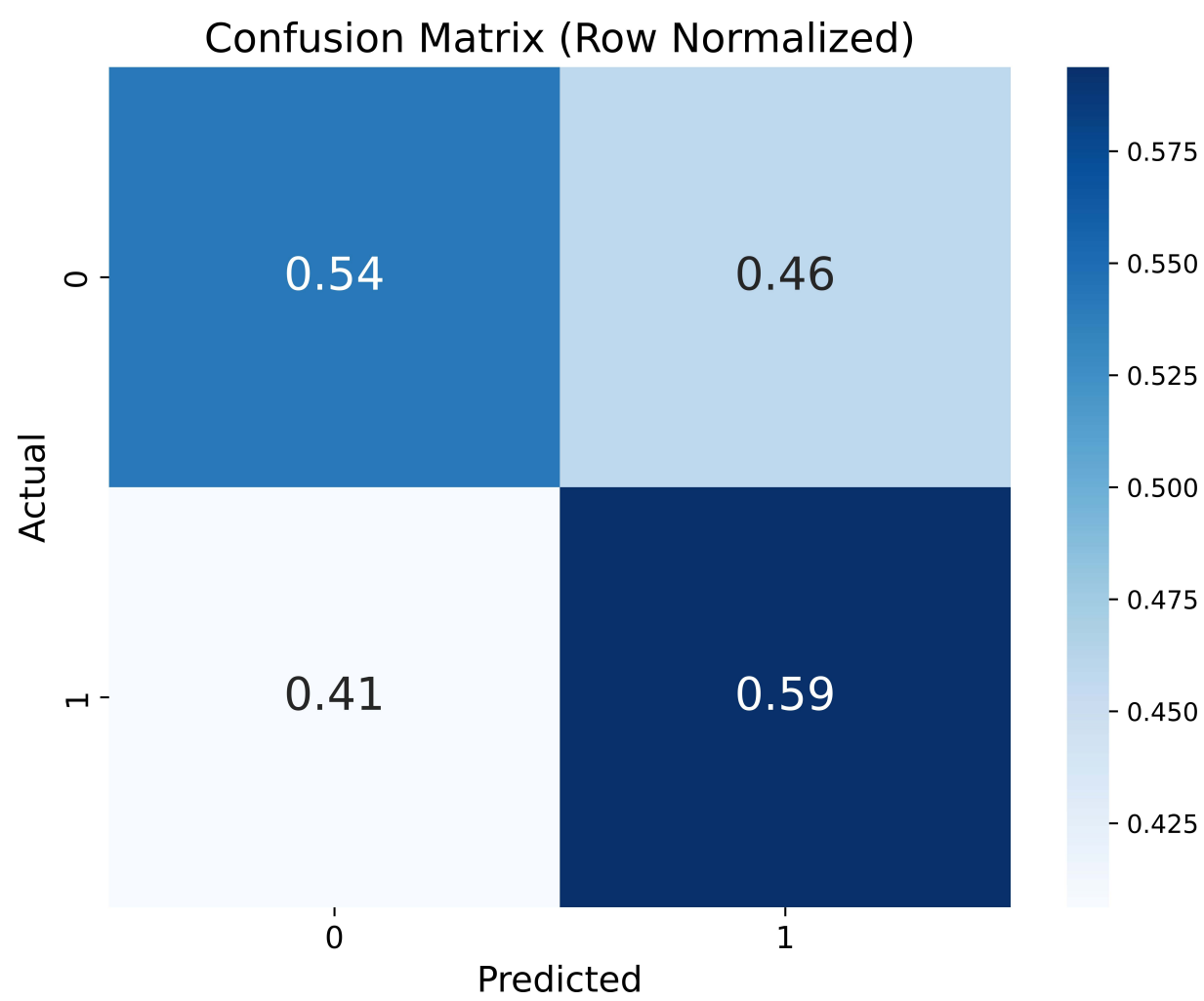

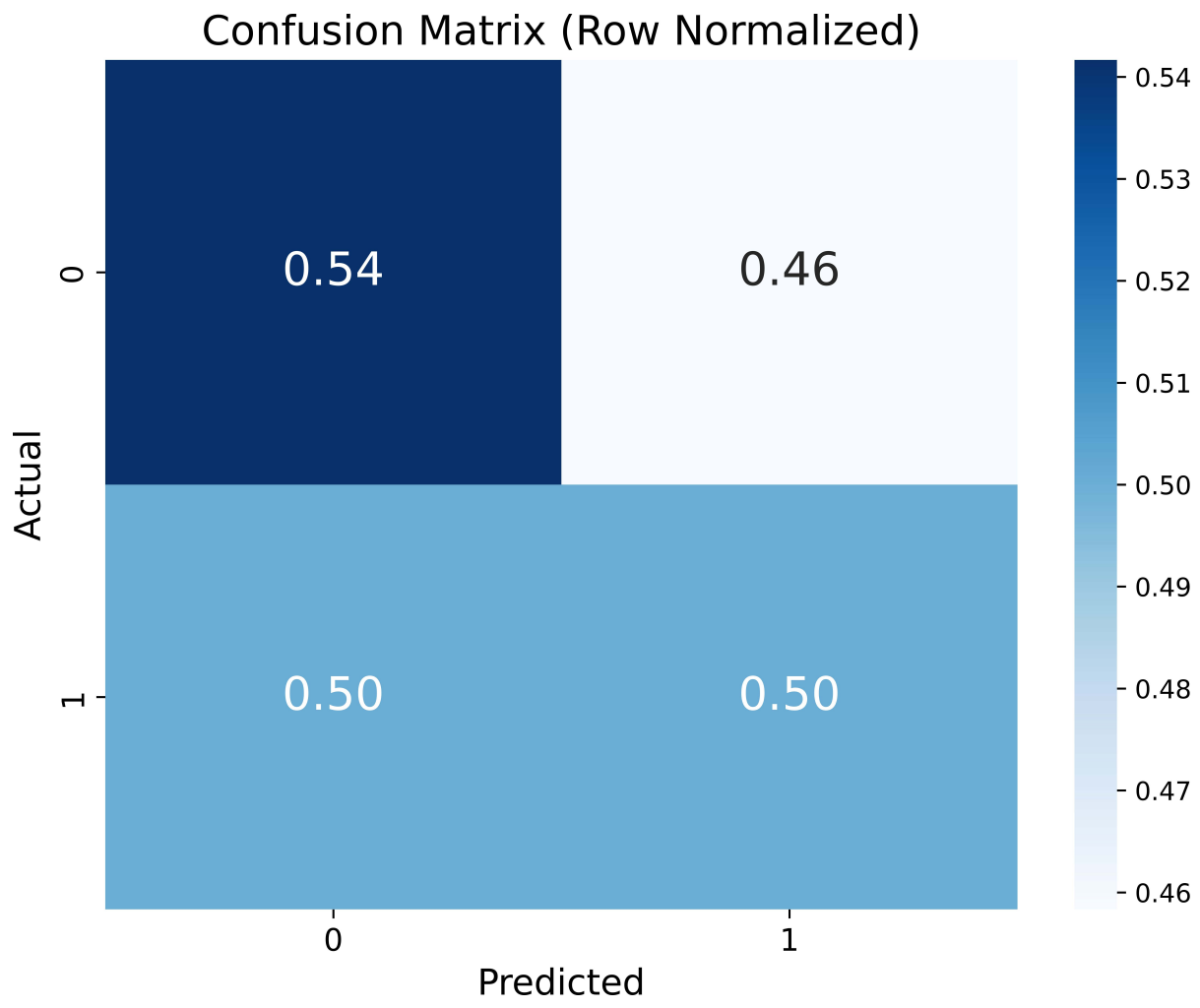

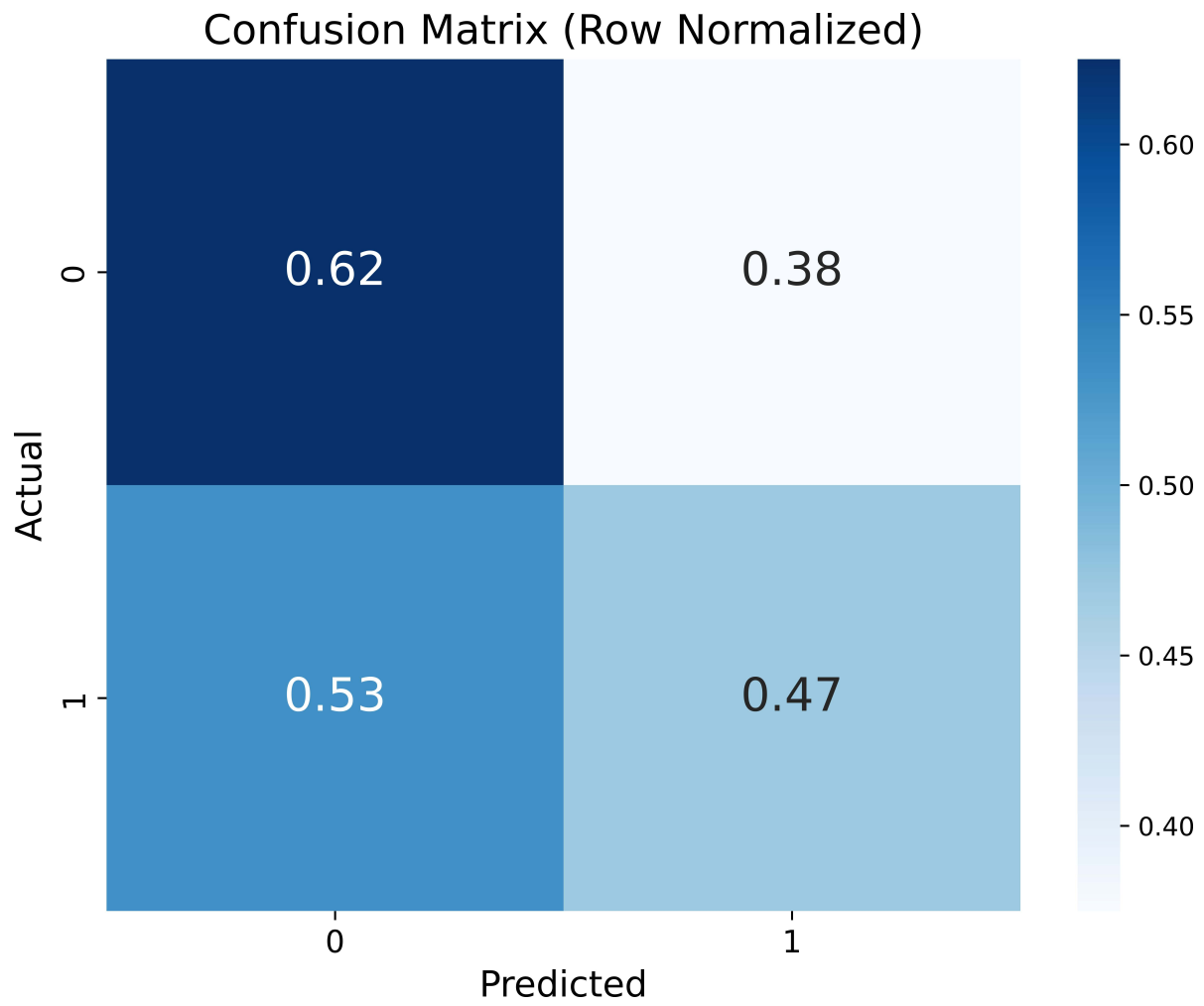

© 2026 The Author(s). Published by Bentham Science Publisher.

This is an open access article distributed under the terms of the Creative Commons Attribution 4.0 International Public License (CC-BY 4.0), a copy of which is available at: https://creativecommons.org/licenses/by/4.0/legalcode. This license permits unrestricted use, distribution, and reproduction in any medium, provided the original author and source are credited.

HOW TO CITE:
Jiang H, Zhu B, Xia L, Han Y. Non-invasive Prediction of Lung Cancer Histological Differentiation *via* Radiomics and Multi-binary Classification Models. Curr Med Imaging, 2026; 22: e15734056415019.
http://dx.doi.org/10.2174/0115734056415019251016111351
